# Mindfulness Practice and Increases in Gray Matter Density, Gray Matter Volume, and Cortical Thickness: A Scoping Review

**DOI:** 10.3390/brainsci16050483

**Published:** 2026-04-30

**Authors:** Colin Rafter, Erika McCarthy, Curt Stilp, Jason Brumitt

**Affiliations:** 1 Doctor of Medical Science Program, College of Medical Science, George Fox University, Newberg, OR 97132, USA; crafter23@georgefox.edu (C.R.); emccarthy@georgefox.edu (E.M.); cstilp@georgefox.edu (C.S.); 2 School of Physical Therapy, College of Allied Health, George Fox University, Newberg, OR 97132, USA

**Keywords:** brain morphology, hippocampus, meditation, mindfulness-based interventions, neuroplasticity

## Abstract

**Highlights:**

**What are the main findings?**

- Mindfulness practice, increasingly integrated into both clinical and wellness settings, has been associated with structural changes in brain regions tied to cognitive control, interoception, self-referential processing, attention, memory, and emotional regulation.
- Evidence from eight of the nine studies included in this review indicates that the practice is associated with increases in gray matter density, gray matter volume, and cortical thickness.

**What are the implications of the main findings?**

- Mindfulness practice appears to hold promise as a practical, easy to access, low-cost tool for promoting brain health through associated structural change.
- Given that mindfulness has the potential to mitigate cognitive decline and buffer against psychological hardship related to stress, anxiety, or depression, further high-quality research is certainly warranted.

**Abstract:**

**Background/Objectives**: Mindfulness-based interventions (MBIs) have been linked to psychological and cognitive benefits, yet evidence for their impact on brain structure remains sparse. Neuroimaging suggests MBIs may alter gray matter volume (GMV), density (GMD), and cortical thickness (CT). The purpose of this scoping review was to investigate structural neuroplasticity following MBIs. **Methods**: Following PRISMA-ScR guidelines, databases were searched for studies published between 2010 and 2023 that used structural MRI to assess structural brain changes in subjects after receiving MBIs. Nine studies met inclusion criteria, including five randomized controlled trials. **Results**: Mindfulness interventions ranged from 10 h of training to long-term practice spanning decades. Structural changes were most consistently observed in the insula, prefrontal cortex, hippocampus, posterior cingulate, and temporoparietal junction, regions tied to interoception, executive control, and self-referential processing. The greatest structural changes were reported in studies implementing multi-month interventions or long-term meditative practice. **Conclusions**: MBIs are associated with structural brain changes across a limited and heterogeneous body of literature, but current evidence is insufficient to draw firm conclusions regarding causality or consistency of effect. Larger, diverse, and more methodologically rigorous trials with extended follow-up are needed to clarify the durability and significance of observed changes.

## 1. Introduction

Mindfulness, an ancient practice rooted in the observation and acceptance of the present moment, has recently gained significant traction as a non-pharmacological-based approach to improving mental health and cognitive function [1,2,3]. Originally rooted in Buddhist tradition, the practice has found a foothold in modern medicine and is now regularly incorporated into clinical wellness programs [2,3]. Not only is the practice currently being utilized by healthcare providers as a tool, it is also used in education and occupational settings [2,3]. The integration of mindfulness practices into these environments is driven by mounting evidence suggesting it has beneficial effects on emotional regulation, attention, resilience to stress, and cognitive function [1,2,3,4]. As the popularity of the practice has grown, so has the interest in the neurobiological effects of it. Researchers have started exploring how these practices, specifically mindfulness-based interventions (MBIs), might affect brain structure, with a particular focus on gray matter volume (GMV), gray matter density (GMD), and cortical thickness (CT).

MBIs are structured programs designed to systematically teach mindfulness practices in standardized and reproducible formats. The most widely studied MBIs include mindfulness-based stress reduction (MBSR), integrative body–mind training (IBMT) and mindfulness-based cognitive therapy (MBCT), which were developed to translate contemplative practices into clinically applicable protocols [2,5]. These interventions are typically delivered over several weeks and have been applied across a variety of clinical and non-clinical populations, including individuals with anxiety, depression, chronic pain, and healthy adults. MBIs have been increasingly studied for their potential to induce both functional and structural changes in the brain, making them a relevant focus for neuroimaging research [2,5].

Over the past two decades, researchers have examined the structural and functional neural correlates of mindfulness practice [6]. Structural brain changes, particularly in GMV, GMD, and CT, have been hypothesized to be elicited through neuroplastic mechanisms induced by mindfulness practice [1,5,7,8,9,10,11,12,13]. Neuroplasticity, which is the brain’s ability to adapt and restructure itself by forming new neural connections throughout someone’s life, is thought to be the mechanism through which mindfulness intervention induces many of the behavioral changes [1,5,7,8,9,10,11,12,13]. Neuroimaging studies of mindfulness practice have consistently implicated several regions that are involved in the brain’s restructuring, including the dorsolateral prefrontal cortex (PFC), associated with attentional control; the anterior cingulate cortex (ACC), implicated in emotion regulation; the hippocampus, involved in memory consolidation; the insula, associated with interoceptive awareness; and the posterior cingulate cortex (PCC), linked to self-referential processing [1,4,5,7,8,9,10,11,12,13,14].

If mindfulness can increase GMV, GMD, or CT via neuroplasticity, advancing the understanding of this relationship may have important clinical implications, not only for enhancing emotional regulation and behavioral outcomes, but also for its potential role in supporting brain health. However, whether MBIs confer long-term neuroprotective effects remains an open question and an area for future research. As a low-risk, low-cost intervention, mindfulness offers a promising avenue for promoting brain health. Preliminary evidence suggests that mindfulness practice may induce measurable structural brain changes in regions associated with attention, emotional regulation, and self-referential processing [1,2,4,15]. The purpose of this scoping review is to gather and synthesize the existing body of evidence from RCTs, as well as complementary studies, that examines the impact of mindfulness practice on brain morphology. Existing studies are characterized by variability in intervention type, duration, and neuroimaging methodology, as well as differences in participant characteristics such as prior meditation experience and baseline behavioral or cognitive measures. In addition, findings across studies are often inconsistent, and many are limited by small sample sizes and methodological heterogeneity. A scoping review approach was therefore selected to map the current literature, identify patterns in reported structural changes, and highlight gaps to guide future research.

## 2. Materials and Methods

A literature search was conducted using PubMed and Medline to identify studies examining structural brain changes associated with mindfulness practice. The following keywords were searched: mindfulness, meditation, brain, brain changes, gray matter, gray matter volume, gray matter density, cortical thickness, structure changes, and structural brain changes. While additional databases such as PsycINFO and Embase may have yielded relevant studies, their exclusion represents a potential limitation, particularly given the multidisciplinary nature of mindfulness research, and may have resulted in missed studies.

A scoping review was determined to be the appropriate study design to identify research relevant to this topic. The review was conducted in accordance with the Preferred Reporting Items for Systematic Reviews and Meta-Analyses extension for Scoping Reviews (PRISMA-ScR) framework (in Supplementary Materials), which guided the design, reporting, and synthesis processes. This scoping review was registered with the Open Science Framework (DOI: 10.17605/OSF.IO/VT72Y). Registration was completed prior to data collection to ensure transparency and replicability.

**Figure 1:**
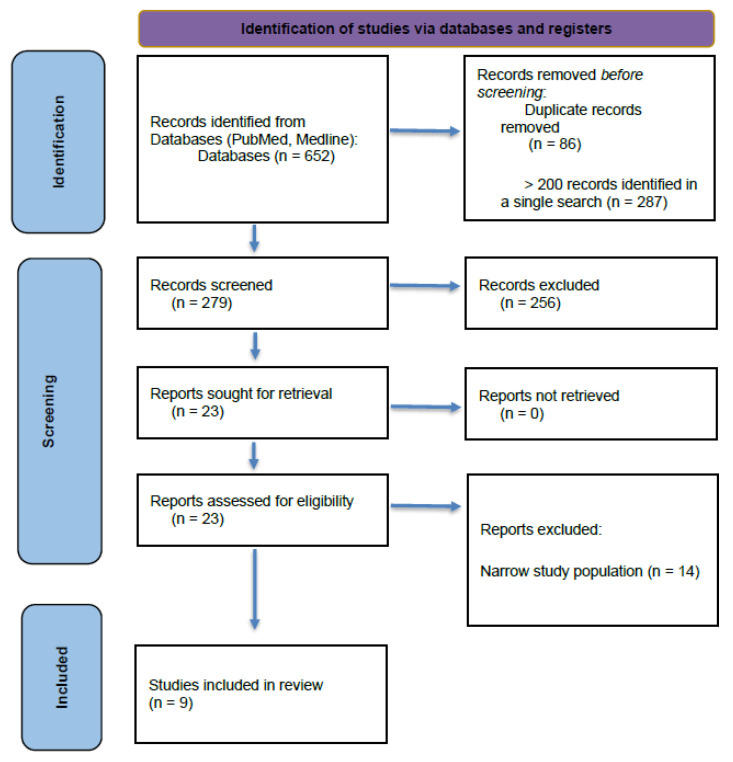
PRISMA flow diagram. “Narrow study population” refers to studies that focused on highly specific or non-generalizable populations (e.g., disease-specific cohorts or specialized subgroups) that were not aligned with the broader inclusion criteria of the review.

If a search strategy yielded fewer than 200 articles, all results were advanced for formal screening. This approach was used to ensure that potentially relevant studies were not prematurely excluded when search terms yielded a limited number of results, consistent with the exploratory nature of a scoping review. The initial screening was conducted by title, with abstracts from pertinent studies reviewed to determine potential inclusion. Abstract evaluation was also performed in instances where the study’s applicability could not be ascertained from the title alone. The primary investigator (C.R.) reviewed each article to determine eligibility for inclusion in the study. Table 1 displays the keyword or keyword combinations utilized in the search strategy for this scoping review, and Figure 1 depicts the selection process used to identify and extract the studies that were ultimately included in the review.

The search strategy was intentionally broad to maximize sensitivity and capture all potentially relevant studies examining structural brain changes associated with mindfulness practice. Keywords were iteratively refined and combined using Boolean operators to balance sensitivity and specificity. Given the variability in terminology used across neuroimaging and mindfulness research, multiple keyword combinations were employed to ensure comprehensive coverage of the literature. 

### 2.1. Inclusion and Exclusion Criteria

Studies were eligible if they were published in English in peer-reviewed journals between 2010 and the present, involved adults (>18 years), and used a quantitative design (e.g., RCT, controlled longitudinal, pre-post, or cross-sectional matched) to evaluate a MBI such as mindfulness-based stress reduction (MBSR) or integrative body—mind training (IBMT) with pre- and post-intervention MRI measures of GMV, GMD, or CT. Exclusion criteria: Studies were excluded if they were non-quantitative, pediatric, lacked a mindfulness component, relied solely on functional imaging, or were reviews, qualitative reports, case studies, dissertations, or conference abstracts.

### 2.2. Neuroimaging Analysis Techniques

Across the included studies, a variety of neuroimaging techniques were employed to quantify structural brain changes associated with mindfulness practice. The techniques included voxel-based morphometry (VBM), surface-based morphometry (SBM), CT analysis, and region-of-interest(ROI) morphometry.

Voxel-based morphometry is a whole-brain, voxel-wise technique that utilizes high-resolution T1-weighted MRI scans to detect differences in local concentrations of GM by statistically comparing each voxel across participants [7,8,9,10]. VBM is incredibly sensitive to miniscule volumetric changes, though it may be influenced by spatial normalization and smoothing parameters [6,7,8,9,10]. SBM is a technique that focuses on the cortical surface, reconstructing and analyzing geometric properties such as surface area and cortical folding patterns [7,9]. SBM allows more precise alignment of cortical features across subjects and can capture changes not readily identified by VBM [7]. CT analysis is a specific surface-based metric that measures the distance between the white matter and pial surfaces of the cortex, at the gray/white matter junction, across thousands of points [11,13,16]. This measurement modality is particularly sensitive to changes in the cortical mantle, a region that is especially reflective of neuroplastic processes [11,13,16]. ROI morphometry is a technique that utilizes targeted analysis to quantify structural measures, like volume, density, and thickness, within predefined anatomical regions [4,6,12,16]. ROI approaches allow hypothesis-driven investigation of specific brain structures, like the hippocampus or insula, but will miss any changes occurring outside the selected areas [4,6,9,12,16].

The focus on GMV, GMD, and CT in this review was based on their widespread use as primary structural MRI outcomes in studies evaluating structural neuroplasticity. These measures provide complementary information about brain morphology, with GMV and GMD reflecting volumetric and density-based changes, and CT capturing alterations in cortical architecture. Other structural measures, such as surface area or subcortical volumetrics, were not included due to their less consistent reporting in the literature and to preserve comparability across included studies. By focusing on these commonly reported metrics, this review aimed to synthesize findings using the most standardized and comparable indicators of structural brain change.

## 3. Results

Nine studies met the inclusion criteria for this scoping review. The included articles were reviewed by each author, with CR and JB extracting data included in Table 2. Of the nine studies, five are RCTs, two are longitudinal studies, one is a cross-sectional study, and one is a single-group pilot study. Trials examined the effects of MBIs on structural brain changes, focusing on GMV, GMD, and CT [6,7,8,9,10,11,12,13,16]. The interventions varied in type and duration, including traditional MBSR [6,7,11], IBMT [12], multi-modular compassion and attention training [13], the Mindfulness Awareness Program (MAP) [8,16], focused attention meditation (FAM) [10], and long-term meditation practice [9]. Participants ranged from healthy adults to older adults with mild cognitive impairment(MCI), and were recruited across North America, Europe, and Asia [7,8,9,11,12,13,16]. Sample sizes ranged from 6 to 445, with intervention durations ranging from 10 total hours of mindfulness practice over 5 days, to 9 months of regular practice, to long-term meditators (average practice of 20 years) [6,7,8,9,10,11,12,13,16]. This heterogeneity in intervention type and duration, along with differences in participant characteristics such as meditation experience, likely contributes to the variability in observed structural outcomes and should be considered when interpreting patterns across studies.

### Patterns of Structural Brain Change Across Studies

Analysis of the included studies revealed consistent neuroanatomical patterns suggesting that specific brain regions may be particularly sensitive to MBIs [6,8,9,10,11,12,13,16]. For example, the insula, which was structurally changed in three of the reviewed studies, emerged as a commonly involved area [10,11,13]. PFC changes were identified in four of the reviewed studies [8,10,13,16]. The PCC, targeted in two of the studies, reflects engagement of the default mode network and self-referential thought processes during mindfulness programs [6,12]. Taken together, these findings suggest that mindfulness practice may exert its neuroplasticity modulated effects through a distributed network of brain regions involved in attention, emotion regulation, interoception, and self-referential processing, highlighting potential neurobiological pathways through which mindfulness contributes to cognitive and emotional outcomes [1,2,3].

Table 2 summarizes the main outcomes for each study included in this scoping review.

## 4. Discussion

The findings of the nine studies included in this review represent a limited and heterogeneous body of evidence, with both consistent and differing structural outcomes reported across studies and should be interpreted with caution. While the results generally tend to suggest that mindfulness practice has the potential to influence structural changes in GMV, GMD, and CT, the magnitude, location, and direction of these changes does vary between studies [6,8,9,10,11,12,13,16]. Differing study design, participant demographics, and analytic methods might play a part in the discrepancies. Intervention duration could also be a significant factor in the differences observed, as duration was shown to have some significance in the Yu et al. study that did measure structural changes at more than one interval [16]. The small number of included studies, combined with variability in intervention design and participant characteristics, limits the generalizability of these findings and underscores the need for larger, more standardized investigations.

Several studies reported increases in CT or GMV in the insula, PFC, and the ventral PCC, all of which are brain regions associated with interoception, attention, and emotional regulation [11,12,13]. For instance, Santarnecchi et al. found CT increases in the right insula that correlated with reductions in alexithymia and anxiety, while Tang et al. found GMV increases in the ventral PCC following IBMT [11,12]. Furthermore, Valk et al. demonstrated module-specific changes in CT, suggesting that mindfulness-related neuroplasticity may not be uniform but instead depends on the specific attentional or emotional processes being employed within a given practice [13]. Ultimately, the PFC changes identified by Valk, Yu, Lenhart, and Kurth et al. reinforce the PFC’s role in emotional regulation, attentional control, and executive functioning, suggesting that these processes may be strengthened through mindfulness practice [8,10,13,16].

Conversely, Kral et al. performed a large-scale RCT with rigorous methodology, and reported null macrostructural brain effects [7]. This discrepancy may reflect differences in statistical power, study design rigor, or the possibility that smaller studies are more likely to detect or report significant effects. Their findings suggest that 8-week-long MBIs may not be sufficient enough to elicit detectable brain changes at the group level, or that the structural changes are too subtle to measure and are individually unique [7]. This possible aspect of heterogeneity is further supported by the findings of Yu et al., which showed increases and decreases in CT across different brain regions in older adults with MCI [16]. Additionally, some of their findings were transient within the 3-month window, but persistent within the 9-month window [16], further supporting that length of intervention could be a significant factor. This sentiment was also touched on by Kurth et al., whose cross sectional study found that long-term meditators had significantly reduced OFC atrophy when compared to non-meditators [9]. This finding should be interpreted as hypothesis-generating rather than confirmatory, and given the limited and heterogeneous evidence base, the potential for long-term neuroprotective effects remains an emerging and currently untested research question.

Collectively, these studies suggest that MBIs are associated with structural differences in brain regions involved in attention, memory, interoception, self-referential processing, and emotional regulation, although the evidence remains limited by methodological heterogeneity and the small number of eligible studies [6,7,8,9,10,11,12,13,16]. The variability in findings across studies likely reflects a combination of factors, including differences in intervention duration, intensity, baseline participant characteristics, and neuroimaging methodology. Importantly, this heterogeneity limits direct comparability across studies and constrains the ability to draw unified or pooled conclusions. Differences in prior meditation experience and intervention dosage may act as moderating factors that influence the presence, magnitude, and direction of observed structural changes. Brain regions such as the insula, PFC, and PCC were more consistently implicated, which may relate to their roles in interoception, attentional control, and self-referential processing, all of which are central targets of MBIs. In contrast, null or inconsistent findings in other regions may reflect insufficient intervention duration, variability in study design, or limitations in measurement sensitivity. Longer-duration interventions and studies involving experienced meditators were more likely to demonstrate detectable structural differences, suggesting a possible dose–response relationship that remains incompletely characterized. These findings highlight the need for larger, dose-variable RCTs with standardized structural MRI protocols [5,6,7,8,9,10,11,12,13,16,17]. The potential for long-term neuroprotective effects remains an underexplored area that warrants further investigation in larger, longitudinal studies. Future research would also benefit from greater coordination across research teams, including the use of standardized intervention protocols and harmonized neuroimaging methodologies to improve comparability and reproducibility of findings.

### Limitations

Limitations identified across the included studies limit the strength of the findings identified by the authors regarding the association between mindfulness practice and neuroplasticity-mediated brain changes. Lack of dose–response gradients, a common limitation that was found in every study included in this review, hindered any ability to evaluate the effect of frequency/quantity of practice within each study [6,7,8,9,10,11,12,13,16]. Another limitation found throughout the included studies was the lack of additional follow-ups. All follow-ups were conducted at the intervention’s completion, which prevents any conclusions about the persistence of any observed effects [6,8,9,10,11,12,13,16]. Several studies were limited by small sample size and homogenized demographics, which take away from the generalizability and strength of the findings [6,8,10,11,12]. The studies from Yu et al. and Valk et al. were limited by high attrition rates, specifically with MRI acquisition, which reduced the effective sample size and inhibited long-term comparisons [13,16]. Study design limitations were also a common finding, specifically the non-RCT studies and those without active controls or without controls at all [8,10,11]. Even when active controls were included in a study, overlapping circumstantial variables within a participant’s personal life may have influenced the findings.

In addition to the limitations within the body of evidence presented above, this scoping review itself has several limitations. Screening, eligibility assessment, and data extraction were conducted by a single reviewer, which increases the risk of selection bias and potential errors in study inclusion. This approach was used due to project resource constraints. To mitigate this limitation, predefined inclusion and exclusion criteria were established prior to screening to improve consistency in study selection. However, each co-investigator also reviewed included articles to ensure they met eligibility criteria. Additionally, the synthesis of the data was strictly limited to narrative and descriptive review. No statistical analysis was conducted, which limits the ability of this review to generate any quantifiable data regarding pooled effect sizes across the group of included studies.

## 5. Conclusions

Mindfulness practice, increasingly integrated into both clinical and wellness settings, has been associated with structural changes in brain regions tied to cognitive control, interoception, self-referential processing, attention, memory, and emotional regulation [1,2,6,7,8,9,10,11,12,13,16]. Evidence from eight of the nine studies included in this review indicate that the practice is associated with increases in GMV, GMD, or CT [6,8,9,10,11,12,13,16]. While several RCTs and longitudinal studies demonstrate region-specific increases in CT, or GMV, or GMD, the most robust of them all found no significant macrostructural effects [7].

Mindfulness practice appears to hold promise as a potential tool for promoting brain health through associated structural changes. Given that mindfulness has the potential to mitigate cognitive decline and buffer against psychological hardship related to stress, anxiety, or depression, further high-quality research is certainly warranted. Future studies should aim to identify optimal intervention parameters such as type of mindfulness and duration, determine whether the practice has more benefit within certain populations, and clarify to what extent any observed structural changes mediate improvements in mental/cognitive health and behavioral modulation. Robust randomized trials with long-term follow-ups will be essential to strengthening the evidence base and informing clinical guidelines for the implementation of mindfulness as a cognitive/behavioral tool.

## Figures and Tables

**Table 1:** Search strategy by keyword and number of identified, relevant, and included articles.

Keywords	Identified	Determined as Potentially Relevant	Included in Scoping Review
Mindfulness	33,893	-	-
Mindfulness practice	8455	-	-
Mindfulness practice AND brain	852	-	-
Mindfulness practice AND brain changes	287	-	-
Mindfulness practice AND structural brain changes	44	11	3
Mindfulness AND brain AND structure changes	177	12	5
Mindfulness OR meditation AND brain AND structure changes AND gray matter	37	5	3
Mindfulness OR meditation AND brain AND structure changes AND cortical thickness	23	10	5
Mindfulness AND brain AND structure changes AND gray matter	25	6	5
Mindfulness practice AND gray matter	36	13	4
Mindfulness practice AND gray matter density	4	2	1
Mindfulness practice AND gray matter volume	19	7	1

**Table 2:** Study, methods, and outcomes of the studies.

Author/Year	Study Design	Subjects	Geographical Location	Intervention	MRI Modality	Primary Outcomes	Secondary/Behavioral Outcomes
Kral et al. [7] (2022)	2 pooled RCTs	218 healthy adults	Madison, Wisconsin United States of America	Duration: 8 weeks, follow-up immediately post intervention Intervention: MBSR Comparison/Control: Active Health Enhancement Program (HEP) and waitlist(WL) Outcomes: GMV, GMD, CT changes Outcome Measures: - Structural Changes: SBM for CT, VBM for GMD/GMV - Behavioral Changes: none	VBM, SBM, CT, ROI	No MBSR-specific structural changes vs. controls There were no significant group differences for change in brain structure for MBSR compared to HEP or WL for any of the ROIs (*p* > 0.10) ROI-specific: (insula, hippocampus, amygdala, and caudate) = no significant group differences for change in GMV No significant within-group changes in brain structure across time (*p* > 0.05)	None
Santarnecchi et al. [11] (2014)	RCT	48 healthy adults	Siena, Italy	Duration: 8 weeks, follow-up immediately post intervention Intervention: MBSR (2.5 h weekly, daily home practice, and a retreat) Comparison/Control: Passive control Outcomes: CT changes, behavioral changes (anxiety, worry, alexithymia, depression, mindful attention) Outcome Measures: - Structural Changes: Voxel-Based Cortical Thickness (VBCT) toolbox for Statistical Parametric Mapping (SPM) software - Behavioral Changes: State–Trait Anxiety Inventory (STAI), Beck Depression Inventory II (BDI II), Mindful Attention Awareness Scale (MAAS), Penn State Worry Questionnaire (PSWQ), Toronto Alexithymia Scale (TAS-20)	The VBCT (VBM) toolbox for SPM was used to calculate GM CT	Increase in CT in the right anterior insula and bilateral somatosensory cortices VBCT model (F(1,25) = 5.456; *p* = 0.008; n^2^ = 0.467), indicating an increase in CT greater in the MBSR than in the control group Specifically located in the right insula (F(1,25) = 12.134; *p* = 0.001) and somatosensory cortex (F(1,25) = 10.316; *p* = 0.002) Change in CT in the control group were not significant for either insula (F(1,25) = −1.12; *p* = 0.44) and somatosensory cortex (F(1,25) = 0.78; *p* = 0.65) No clusters of GMV reached the statistical significance threshold for main or interaction effects	Decrease in anxiety, worry, and alexithymia. Insula CT correlated with alexithymia reduction Anxiety: - Mean decrease of ~6.2 points on the STAI Alexithymia decrease: *p* < 0.004 State anxiety decrease: *p* < 0.031 Depression level decrease: *p* < 0.046 MBSR trainees showed a significant pre–post decrease in alexithymia (t(22) = 3.142; *p* < 0.004), worry (t(22) = 2.665; *p* < 0.012), state anxiety (t(22) = 2.259; *p* < 0.031) and depression levels (t(22) = 2.086; *p* < 0.046), while no differences were detected by the MAAS (t(22) = 0.879; *p* < 0.418). No differences were found for participants in the control group (*p* > 0.05 corrected) The rate of changes in CT were correlated with the changes in psychological indexes of interest(Brain/Psy), revealing a significant negative correlation for alexithymia level and insula cluster thickness values (r = −0.712; *p* < 0.01) in MBSR subjects after the training
Tang et al. [12] (2020)	RCT	40 healthy adults	Dalian, China	Duration: 5 days, with 10 h total, immediate follow-up Intervention: 10 h of Integrative body–mind Training (IBMT) Comparison/Control: Relaxation training (RT) Outcomes: GMV changes, mood changes Outcome Measures: - Structural Changes: GMV changes in the PCC, isthmus of cingulate (ISC), retrosplenial cortex (RSC) - Behavioral Changes: Adult Temperament Questionnaire (ATQ)	VBM, ROI	Increase in GMV in the ventral PCC after brief training (5 days, total 10 h) Significant effect on the right ventral PCC/ISC volume F(1,36) = 5.08, *p* = 0.03 IBMT group had a significantly higher % change (M = 0.88, SD = 3:50) relative to the RT group (M = −2.66, SD = 5.47). A large effect size, 0.124 (partial eta-squared), was detected No significant difference was detected in RSC volume between the IBMT and RT group (*p* > 0.05)	Decrease in sadness, as defined by lower level of unpleasant affect, mood, and energy-related to object or person loss, disappointment, and exposure to suffering Sadness was the only one of the four temperamental traits that showed a significantly negative correlation with the right ventral PCC/ISC change (r = −0.535, *p* < 0.018)
Valk et al. [13] (2017)	RCT	Randomized training cohorts, 445 healthy adults	Leipzig, Germany	Duration: 9 months, 3 × 3-month-long modules, with follow-ups at the end of each module Intervention: Modules on presence (mindfulness/attention), affect(compassion/emotion), perspective (theory of mind (ToM)/metacognition) Comparison/Control: Each module served as active control for others, retest cohort Outcomes: CT changes, behavioral changes (domain-specific) Outcome Measures: - Structural Changes: CT, whole brain - Behavioral Changes: EmpaToM task (compassion, ToM), attention test (cued flanker). Compassion was quantified as a mean of compassion ratings across all experimental conditions	CT	Module-specific CT increases - Presence → right PFC and ACC increases FWE < 0.001) - Affect/compassion → Increases in the right insula extending to the temporal pole, with findings consistent across all cohorts undergoing affect training [TC1, T1 → T2 (r = 0.37); TC2, T2 → T3 (r = 0.28); TC3, T0 → T1 (r = 0.31) - Affect/perspective → Bilateral occipital regions extending to inferior temporal cortices (FWE < 0.05 (left) and <0.001 [right]) Increases in thickness in right lateral and medial frontal regions (FWE < 0.001), together with focal decreases in the right lingual gyrus (FWE < 0.025)	Domain-specific behavioral increases tracked: increased attention, affect presence, compassion, ToM
Yu et al. [16] (2021)	RCT	54 older adults w/MCI	Singapore, Singapore	Duration: 9 months, with follow-ups at 3 months and 9 months Intervention: Mindfulness Awareness Program (MAP), weekly sessions × 3 months then monthly sessions × 6 months Comparison: Health Education Program (HEP) Outcomes: CT changes, cognition changes (working memory span, divided attention) Outcome Measures: - Structural Changes: CT of whole brain and specific ROIs - Behavioral Changes: Cognitive assessments, digit span backward and block design tests from the Wechsler Adult Intelligence Scale (WAIS-III), delayed recall and recognition tests from the Rey Auditory Verbal Learning Test(RAVLT), Color Trails Test(CTT), and semantic fluency test	CT, ROI	- Increases in CT in right frontal pole (at 9 months) - Transient increase in CT in the left inferior temporal gyrus (at 3 months) - Decreases in CT in the left ACC The MANOVAs on the CT revealed a significant time * group effect between baseline and ninth months (F(8,12) = 3.03; *p* = 0.041; η^2^_partial_ = 0.67), but not between baseline and third month (F(8,29) = 2.01; *p* = 0.080; η^2^_partial_ = 0.36)	Improved working memory span and divided attention
Lenhart et al. [10] (2020)	Longitudinal pre–post	27 healthy adults	Innsbruck, Austria	Duration: 7.3 week average, follow-up immediately after intervention Intervention: Focused attention meditation (FAM) (14 guided session over 7 weeks) + daily home practice Comparison/Control: None (within subject) Outcomes: GM changes, WM microstructure, anxiety, quality of life (QOL) Outcome Measures: - Structural Changes: VBM for GM and DWI for FA - Behavioral Changes: Short-form health survey (SF-36), German version of the State–Trait Anxiety Inventory (STADI)	VBM	Within-group comparison of the 27 meditation participants showed increased GM volumes at follow-up compared to the baseline time-point in clusters of the following: The anterior insula (*p* = 0.001) The inferior frontal gyrus (*p* = 0.001) The caudate nucleus with adjacent regions of the putamen (*p* = 0.002) The superior frontal gyrus (*p* = 0.003) In each case, bi-hemispheric and middle spreading to the superior temporal gyrus (*p* < 0.001) and the cerebellum (*p* = 0.004) on the right side were observed Significant GM decreases were revealed in the inferior parietal lobule as well as the superior and middle temporal gyrus (*p* < 0.001) and the inferior frontal gyrus (*p* = 0.001) on both sides, as well as in the mPFC (*p* < 0.001) and the parahippocampal gyrus spreading to the fusiform gyrus (*p* = 0.001) on the right side Additionally, GM decreases were found in the PCC at height thresholds set to *p* < 0.01 (*p* < 0.001) No significant sex differences were found at height thresholds set to *p* < 0.01	Decrease in state anxiety associated with MCC/PCC and mPFC changes Longitudinal analysis of the questionnaires revealed significant improvements for excitement(*p* = 0.012), concern (*p* = 0.012) and anxiety (*p* = 0.004) as well as psychological well-being (*p* = 0.004) Improved scores of the STADI questionnaire
Kurth et al. [8] (2014)	Single-group pilot	6 healthy older adults	Los Angeles, California, United States of America	Duration: 6 weeks, follow-up immediately after intervention Intervention: Mindfulness Awareness Practices (MAPs), 6 weekly 2 h session Comparison/Control: None (within subject) Outcomes: GM changes Outcome Measures: - Structural Changes: VBM - Behavioral Changes: none	VBM	Significant increases in GM in right precuneus. Decreases in GM in left PFC, right hippocampus, right thalamus, and right parietal cortex VBM analysis revealed one cluster indicating significant GM increase in the right precuneus (247 voxels, cluster maximum [x;y;z]: 6;−64;19, *p* = 2.4 × 10^−15^) and decreases in the left PFC (408 voxels, x;y;z: −39;50;−8, *p* = 1.0 × 10^−10^), right hippocampus (136 voxels, x;y;z: 24;−36;3, *p* = 1.3 × 10^−7^), right thalamus (199 voxels, x;y;z:3;−21;12, *p* = 1.7 × 10^−5^), and right parietal cortex (311 voxels, x;y;z: 9;−45;58, *p* = 1.8 × 10^−15^)	n/a
Kurth et al. [9] (2023)	Cross-sectional matched	100 healthy adults: 50 long-term meditators and 50 control participants	Berlin, Germany	Duration: ~20-year practice; cross-sectional analysis Intervention: Long-term meditation practice Comparison/Control: Matched non-meditators Outcomes: Orbitofrontal cortex (OFC) overall tissue loss rate (healthy aging) Outcome Measures: - Structural Changes: ROI morphometry (OFC Fo1-Fo7) - Behavioral Changes: none	ROI	Reduced age-related OFC atrophy in meditators; multiple Fo subregions significant(OFC is made up of 7 (Fo1–Fo7) defined subregions) Significant group-by-age interaction for the left and right OFC, as well as for left and right Fo2, Fo3, Fo4, and Fo7. In addition, significant group-by-age interactions were observed for left Fo5 and right Fo6. Effect sizes (calculated as Cohen’s d) ranged between d = 0.374 and d = 0.806, suggesting a range from small to large effects.	Supports neuroprotection and healthy aging hypothesis
Hölzel et al. [6] (2011)	Controlled longitudinal	33 healthy adults: 16 in MBSR and 17 in WL	Boston, Massachusetts United States of America	Duration: 8 weeks, follow-up immediately post intervention Intervention: MBSR daily, ~27 min Comparison/Control: Waitlist Outcomes: GMD changes, mindfulness facets (behavioral attributes) Outcome Measures: - Structural Changes: VBM of whole brain and in ROI of hippocampus - Behavioral Changes: Five Facet Mindfulness Questionnaire (FFMQ)	VBM, ROI	Increases in GMD in left hippocampus Increases in GMD in PCC, TPJ, cerebellum (whole brain) ROI: MBSR group identified a small cluster in the left hippocampus with increased gray matter concentration (peak voxel MNI coordinates x,y,z: −36,−34,−8; t(15) = 6.89; voxel level *p* = 0.014, corrected for multiple comparisons with FWE correction; cluster size k = 30 Whole brain: Cluster located in four regions (PCC; left temporo-parietal junction (TPJ); and two clusters in the cerebellum) Group × time interactions were significant for all 4 regions, indicating that increases in GM concentration were significantly greater in the MBSR than in the control group: PCC (F(1,29) = 50.124; *p* = 0.001), TPJ (F(1,29) = 11.456; *p* = 0.002), cerebellar vermis/brainstem (F(1,29) = 11.292; *p* = 0.002), lateral cerebellum (F(1,29) = 9.806; *p* = 0.004)	MBSR participants significantly increased their mindfulness facet scores (acting with awareness, observing, non-judgmental attitude) Confirmed significant group/time interactions for three of the five mindfulness subscales: - Acting with awareness: F(1,26) = 16.87, *p* < 0.001 - Observing: F(1,26) = 7.09, *p* = 0.013 - Non-judging: F(1,26) = 4.61, *p* = 0.041 - Describing: F(1,26) = 1.95, *p* = 0.175 - Non-reactivity: F(1,26) = 2.79, *p* = 0.107 Significant gains in the MBSR group: - Acting with awareness: t(13) = 3.665, *p* = 0.003 - Observing: t(13) = 4.218, *p* = 0.001 - Non-judging: t(13) = 3.580, *p* = 0.003

## Data Availability

No new data were created or analyzed in this study.

## Supplementary Materials

The following supporting information can be downloaded at: https://www.mdpi.com/article/10.3390/brainsci16050483/s1. Reference [18] are cited in the Supplementary Materials.

## Abbreviations

The following abbreviations are used in this manuscript:

ACC	Anterior cingulate cortex
CT	Cortical thickness
GMD	Gray matter density
GMV	Gray matter volume
FAM	Focused Attention Meditation
IBMT	Integrative body–mind training
MAP	Mindfulness Awareness Program
MBI	Mindfulness-based interventions
MBSR	Mindfulness-based stress reduction
MCI	Mild cognitive impairment
PCC	Posterior cingulate cortex
PFC	Prefrontal cortex
PRISMA-ScR	Preferred Reporting Items for Systematic Reviews and Meta-Analyses extension for Scoping Reviews
ROI	Region-of-interest
RCT	Randomized controlled trial
SBM	Surface-based morphometry
VBM	Voxel-based morphometry
